# Effects of abiotic stress on plants: a systems biology perspective

**DOI:** 10.1186/1471-2229-11-163

**Published:** 2011-11-17

**Authors:** Grant R Cramer, Kaoru Urano, Serge Delrot, Mario Pezzotti, Kazuo Shinozaki

**Affiliations:** 1 Department of Biochemistry and Molecular Biology, Mail Stop 330, University of Nevada, Reno, Nevada 89557, USA; 2 Gene Discovery Research Group, RIKEN Plant Science Center, 3-1-1 Koyadai, Tsukuba 305-0074, Japan; 3 Univ. Bordeaux, ISVV, Ecophysiologie et Génomique Fonctionnelle de la Vigne, UMR 1287, F-33882 Villenave d'Ornon, France; 4 Dipartimento di Biotecnologie, Università di Verona, Strada le Grazie 15, 37134 Verona, Italy

## Abstract

The natural environment for plants is composed of a complex set of abiotic stresses and biotic stresses. Plant responses to these stresses are equally complex. Systems biology approaches facilitate a multi-targeted approach by allowing one to identify regulatory hubs in complex networks. Systems biology takes the molecular parts (transcripts, proteins and metabolites) of an organism and attempts to fit them into functional networks or models designed to describe and predict the dynamic activities of that organism in different environments. In this review, research progress in plant responses to abiotic stresses is summarized from the physiological level to the molecular level. New insights obtained from the integration of omics datasets are highlighted. Gaps in our knowledge are identified, providing additional focus areas for crop improvement research in the future.

## Reviews

Recent advances in biotechnology have dramatically changed our capabilities for gene discovery and functional genomics. For the first time, we can now obtain a holistic "snapshot" of a cell with transcript, protein and metabolite profiling. Such a "systems biology" approach allows for a deeper understanding of physiologically complex processes and cellular function [1]. New models can be formed from the plethora of data collected and lead to new hypotheses generated from those models.

Understanding the function of genes is a major challenge of the post-genomic era. While many of the functions of individual parts are unknown, their function can sometimes be inferred through association with other known parts, providing a better understanding of the biological system as a whole. High throughput omics technologies are facilitating the identification of new genes and gene function. In addition, network reconstructions at the genome-scale are key to quantifying and characterizing the genotype to phenotype relationships [2].

In this review, we summarize recent progress on systematic analyses of plant responses to abiotic stress to include transcriptomics, metabolomics, proteomics, and other integrated approaches. Due to space limitations, we try to emphasize important perspectives, especially from what systems biology and omics approaches have provided in recent research on environmental stresses.

## Plant responses to the environment are complex

Plants are complex organisms. It is difficult to find an estimate of the total number of cells in a plant. Estimates of the number of cells in the adaxial epidermal layer and palisade mesophyll of a simple Arabidopsis leaf are approximately 27,000 and 57,000 cells, respectively [3]. Another estimate of the adaxial side of the epidermal layer of the 7^th ^leaf of Arabidopsis was close to 100,000 cells [4] per cm^2 ^of leaf area. An Arabidopsis plant can grow as large as 14 g fresh weight with a leaf area of 258 cm^2 ^(11 g fresh weight) [5]. Thus, we estimate that a single Arabidopsis plant could have approximately 100 million cells (range of 30 to 150 million cells assuming 2.4 to 11 million cells per g fresh weight). A one million Kg redwood tree could possibly have 70 trillion cells assuming a cell size 100 times larger than an Arabidopsis cell. Combine that with developmental changes, cell differentiation and interactions with the environment and it is easy to see that there are an infinite number of permutations to this complexity.

There is additional complexity within the cell with multiple organelles, interactions between nuclear, plastidial and mitochondrial genomes, and between cellular territories that behave like symplastically isolated domains that are able to exchange transcription factors controlling gene expression and developmental stages across the plasmodesmata. A typical plant cell has more than 30,000 genes and an unknown number of proteins, which can have more than 200 known post-translational modifications (PTMs). The molecular responses of cells (and plants) to their environment are extremely complex.

## Environmental limits to crop production

In 1982, Boyer indicated that environmental factors may limit crop production by as much as 70% [6]. A 2007 FAO report stated that only 3.5% of the global land area is not affected by some environmental constraint (see Table three point seven in http://www.fao.org/docrep/010/a1075e/a1075e00.htm). While it is difficult to get accurate estimates of the effects of abiotic stress on crop production (see different estimates in Table 1), it is evident that abiotic stress continues to have a significant impact on plants based upon the percentage of land area affected and the number of scientific publications directed at various abiotic stresses (Table 1). If anything the environmental impacts are even more significant today; yields of the "big 5" food crops are expected to decline in many areas in the future due to the continued reduction of arable land, reduction of water resources and increased global warming trends and climate change [7].

**Table 1:** Estimates of the impacts of abiotic stresses on crop production and published research.

Stress Type	% of global land area affected*	% of global rural land area affected**	Number of Publications***
**Abiotic Stress**		96.5	**35,363**

**Water**			**4819**
Deficit or Drought	64	16	4137
Flooding or Anoxia	13	10	682

**Temperature**			**9715**
Cold	57	26	3798
Chilling			187
Freezing			350
High or heat			5380

**Light**			**7659**
Low			3081
High			4578

**Chemical/Soil**		50	**12391**
Salt or salinity	6	6	3498
Mineral deficiency or low fertility	9	39	222
Mineral toxicity			437
Acid soil	15		3646
Air pollutants			
Ozone			1369
Sulfur dioxide			378
NO_x _oxide			2001
Elevated CO_2_			840

Miscellaneous (e.g. wind, mechanical, etc.)			**779**

*based on FAO World Soil Resources Report 2000 ftp://ftp.fao.org/agl/agll/docs/wsr.pdf.

** based on Tables three point six and three point seven of 2007 FAO Report http://www.fao.org/docrep/010/a1075e/a1075e00.htm

*** data based on simple searches in PubMed between 2001 and July 7, 2011.

**Figure 1:**
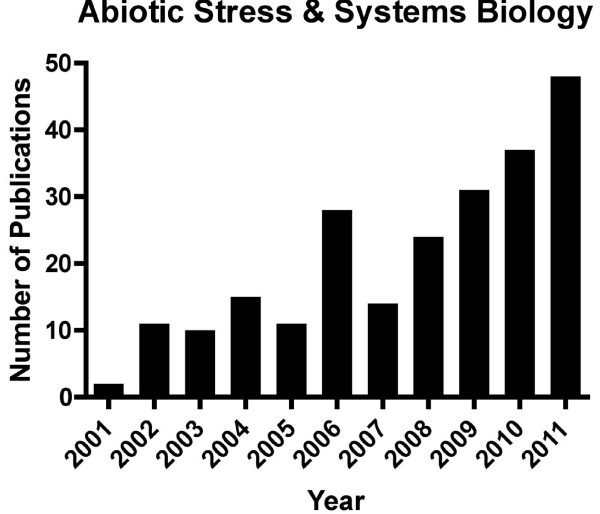
**The number of publications per year related to systems biology and abiotic stress**. Key words used in the search of PubMed included: plant, systems biology, and abiotic stress (including stress sub-terms; e.g. drought or water deficit or dehydration). *The number for the year 2011 was estimated by doubling the 6-month value.

This growing concern is reflected in the increasing number of publications focused on abiotic stresses. For example, since the pivotal review of systems biology by Kitano in 2002 [1], the number of papers published on abiotic stress in plants using a systems biology approach has increased exponentially (Figure 1).

## Multiple factors limit plant growth

Fundamentally, plants require energy (light), water, carbon and mineral nutrients for growth. Abiotic stress is defined as environmental conditions that reduce growth and yield below optimum levels. Plant responses to abiotic stresses are dynamic and complex [8,9]; they are both elastic (reversible) and plastic (irreversible).

The plant responses to stress are dependent on the tissue or organ affected by the stress. For example, transcriptional responses to stress are tissue or cell specific in roots and are quite different depending on the stress involved [10]. In addition, the level and duration of stress (acute vs chronic) can have a significant effect on the complexity of the response [11,12].

Water deficit inhibits plant growth by reducing water uptake into the expanding cells, and alters enzymatically the rheological properties of the cell wall; for example, by the activity of ROS (reactive oxygen species) on cell wall enzymes [8]. In addition, water deficit alters the cell wall nonenzymatically; for example, by the interaction of pectate and calcium [13]. Furthermore, water conductance to the expanding cells is affected by aquaporin activity and xylem embolism [14-17]. The initial growth inhibition by water deficit occurs prior to any inhibition of photosynthesis or respiration [18,19].

The growth limitation is in part due to the fundamental nature of newly divided cells encasing the xylem in the growing zone [20,21]. These cells act as a resistance to water flow to the expanding cells in the epidermis making it necessary for the plant to develop a larger water potential gradient. Growth is limited by the plant's ability to osmotically adjust or conduct water. The epidermal cells can increase the water potential gradient by osmotic adjustment, which may be largely supplied by solutes from the phloem. Such solutes are supplied by photosynthesis that is also supplying energy for growth and other metabolic functions in the plant. With long-term stress, photosynthesis declines due to stomatal limitations for CO_2 _uptake and increased photoinhibition from difficulties in dissipating excess light energy [12].

One of the earliest metabolic responses to abiotic stresses and the inhibition of growth is the inhibition of protein synthesis [22-25] and an increase in protein folding and processing [26]. Energy metabolism is affected as the stress becomes more severe (e.g. sugars, lipids and photosynthesis) [12,27,28]. Thus, there are gradual and complex changes in metabolism in response to stress.

## Central regulators limit key plant processes

The plant molecular responses to abiotic stresses involve interactions and crosstalk with many molecular pathways [29]. Systems biology and omics approaches have been used to elucidate some of the key regulatory pathways in plant responses to abiotic stress.

One of the earliest signals in many abiotic stresses involve ROS and reactive nitrogen species (RNS), which modify enzyme activity and gene regulation [30-32]. ROS signaling in response to abiotic stresses and its interactions with hormones has been thoroughly reviewed [32]. ROS and RNS form a coordinated network that regulates many plant responses to the environment; there are a large number of studies on the oxidative effects of ROS on plant responses to abiotic stress, but only a few studies documenting the nitrosative effects of RNS [30].

**Figure 2:**
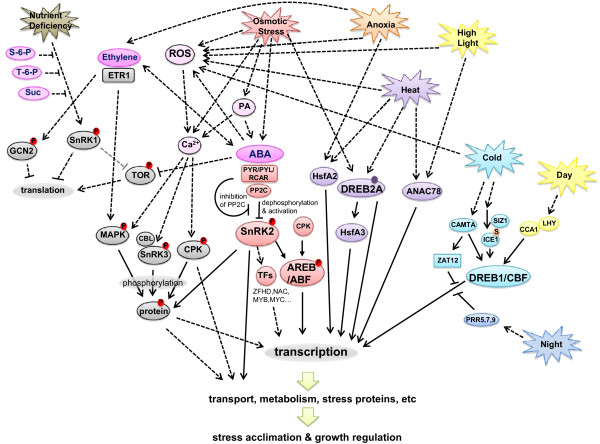
**A simplified working model of a signaling network of plant responses to abiotic stress**. Ovals represent proteins, metabolites or processes. Metabolites have magenta color. Phosphorylated proteins have red circles with a P inside. Sumoylated protein has an orange circle with an S inside. The solid purple circle indicates that DREB2 needs modification to be activated. Solid lines represent direct connections; dotted lines represent indirect connections (acting through some intermediate molecule). The gray line indicates that this reaction has not been shown in plants. Not all linkages and details of stress and hormone effects are shown in this diagram in order to simplify the model. Abbreviations: ABA (abscisic acid), ANAC (Arabidopsis NAC domain-containing protein), CAMTA (calmodulin-binding transcription activator), CBL (calcineurin B-like interacting protein kinase), CCA (circadian clock associated), CPK (calcium-dependent protein kinase), DREB/CBF (dehydration response element binding protein/C-repeat binding factor), ETR1 (ethylene response 1), GCN2 (general control non-repressible 2), HSF (heat shock factor), ICE (inducer of CBF expression), MAPK (mitogen-activated protein kinase), LHY (late elongated hypocotyl), PA (phosphatidic acid), PP2C (protein phosphatase 2C), PRR (pseudo response regulator), PYR/PYL/RCAR (ABA receptors), RNS (reactive nitrogen species), ROS (reactive oxygen species), SIZ (SAP and Miz domain protein), SnRK (sucrose nonfermenting-1 related kinase), TFs (transcription factors), TOR (target of rapamycin), ZAT (zinc finger protein).

Hormones are also important regulators of plant responses to abiotic stress (Figure 2). The two most important are abscisic acid (ABA) and ethylene [33]. ABA is a central regulator of many plant responses to environmental stresses, particularly osmotic stresses [9,34-36]. Its signaling can be very fast without involving transcriptional activity; a good example is the control of stomatal aperture by ABA through the biochemical regulation of ion and water transport processes [35]. There are slower responses to ABA involving transcriptional responses that regulate growth, germination and protective mechanisms.

Recently, the essential components of ABA signaling have been identified, and their mode of action was clarified [37]. The current model of ABA signaling includes three core components, receptors (PYR/PYL/RCAR), protein phosphatases (PP2C) and protein kinases (SnRK2/OST1) [38,39]. The PYR/PYL/RCAR proteins were identified as soluble ABA receptors by two independent groups [38,39]. The 2C-type protein phosphatases (PP2C) including ABI1 and ABI2, were first identified from the ABA-insensitive Arabidopsis mutants *abi1-1 *and *abi2-1*, and they act as global negative regulators of ABA signaling [40]. SNF1-related protein kinase 2 (SnRK2) is a family of protein kinases isolated as ABA-activated protein kinases [41,42]. In Arabidopsis, three members of this family, SRK2D/SnRK2.2, SRK2E/OST1/SnRK2.6, and SRK2I/SnRK2.3, regulate ABA signaling positively and globally, as shown in the triple knockout mutant *srk2d srk2e srk2i *(*srk2dei*)/*snrk2.2 snrk2.3 snrk2.6*, which lacks ABA responses [43]. The PYR/PYL/RCAR - PP2C - SnRK2 complex plays a key role in ABA perception and signaling.

Studies of the transcriptional regulation of dehydration and salinity stresses have revealed both ABA-dependent and ABA-independent pathways [44]. Cellular dehydration under water limited conditions induces an increase in endogenous ABA levels that trigger downstream target genes encoding signaling factors, transcription factors, metabolic enzymes, and others [44]. In the vegetative stage, expression of ABA-responsive genes is mainly regulated by bZIP transcription factors (TFs) known as AREB/ABFs, which act in an ABA-responsive-element (ABRE) dependent manner [45-47]. Activation of ABA signaling cascades result in enhanced plant tolerance to dehydration stress. In contrast, a dehydration-responsive cis-acting element, DRE/CRT sequence and its DNA binding ERF/AP2-type TFs, DREB1/CBF and DREB2A, are related to the ABA-independent dehydration and temperature responsive pathways [44]. DREB1/CBFs function in cold-responsive gene expression [48,49], whereas DREB2s are involved in dehydration-responsive and heat-responsive gene expression [50].

Ethylene is also involved in many stress responses [51-53], including drought, ozone, flooding (hypoxia and anoxia), heat, chilling, wounding and UV-B light [31,33,53]. Ethylene signaling is well defined [51,52], and will not be discussed in detail here. There are known interactions between ethylene and ABA during drought [31], fruit ripening [54,55], and bud dormancy [56]. All of these interactions make the plant response to stress very complex [12,31,52].

In yeast, the well-documented central regulators of protein synthesis and energy are SnRK1 (Snf1/AMPK), TOR1 and GCN2 [57-60]. These proteins are largely controlled by the phosphorylation of enzymes; all three are protein kinases acting as key hubs in the coordination of metabolism during stressful conditions [61]. In plants, TOR activity is inhibited by osmotic stress and ABA [62] and GCN2 activity is stimulated by UV-light, amino acid starvation, ethylene, and cold stress [63]. SnRK1 responds to energy depletion, such as low light, nutrient deprivation or hypoxic conditions [64,65], and interacts with both glucose and ABA signaling pathways [66]. One of the results of this coordinated response is the inhibition of protein synthesis.

Many abiotic stresses directly or indirectly affect the synthesis, concentration, metabolism, transport and storage of sugars. Soluble sugars act as potential signals interacting with light, nitrogen and abiotic stress [67-69] to regulate plant growth and development; at least 10% of Arabidopsis genes are sugar-responsive [68]. Mutant analysis has revealed that sugar signaling interacts with ethylene [70], ABA [71,72], cytokinins [73], and light [74,75]. In grapevine, sugar and ABA signaling pathways interact to control sugar transport. An ASR (ABA, stress-, and ripening-induced) protein isolated from grape berries is upregulated synergistically by ABA and sugars, and upregulates the expression of a hexose transporter [76]. VVSK1, a GSK3 type protein kinase, is also induced by sugars and ABA, and upregulates the expression of several hexose transporters [77].

Stresses such as sugar starvation and lack of light stimulate SnRK1 activity ([64]. Suc-P synthase (SPS), 3-hydroxy-3-methylglutaryl-CoA reductase, nitrate reductase, and trehalose-6-P synthase are negatively regulated by SnRK1 phosphorylation [78], indicating that SnRK1 modulates metabolism by phosphorylating key metabolic enzymes. Post-translational redox modulation of ADPG-pyrophosphorylase, a key control of starch synthesis, by SnRK1 provides an interesting example of interactions between phosphorylation, redox control and sugar metabolism [79]. In Arabidopsis, SnRK1 kinase activity is itself increased by GRIK1 and GRIK2, which phosphorylate a threonine residue of the SnRK1 catalytic subunit [78]. SnRK2 interacts with ABA for the control of stomatal aperture and participates in the regulation of plant primary metabolism. Constitutive expression of SnRK2.6 drastically boosts sucrose and total soluble sugar levels in leaves, presumably by controlling SPS expression [80].

## Systems biology approach to abiotic stress

In the post-genomic era, comprehensive analyses using three systematic approaches or omics have increased our understanding of the complex molecular regulatory networks associated with stress adaptation and tolerance. The first one is 'transcriptomics' for the analysis of coding and noncoding RNAs, and their expression profiles. The second one is 'metabolomics' that is a powerful tool to analyze a large number of metabolites. The third one is 'proteomics' in which protein and protein modification profiles offer an unprecedented understanding of regulatory networks. Protein complexes involved in signaling have been analyzed by a proteomics approach [81,82]. Integration of the different omics analyses facilitates abiotic stress signaling studies allowing for more robust identifications of molecular targets for future biotechnological applications in crops and trees.

## Co-expression analyses identify regulatory hubs

An important application of transcriptomics data is co-expression analysis of target genes using on-line analytical tools, such as ATTED-II (reviewed by [83]). This approach is very promising for understanding gene-gene correlations and finding master genes in target conditions.

In a series of pioneering papers, Hirai et al. [84,85] identified MYB transcription factors regulating glucosinolate biosynthesis in Arabidopsis in response to S and N deficiency using an integrated transcriptomics and metabolomics approach. Genes and metabolites in glucosinolate metabolism were found to be coordinately regulated [84]. Co-expression analysis was used to identify two MYB transcription factors that positively regulate glucosinolate metabolism [85]. Then a knock out mutant and ectopic expression of one of the transcription factors was used to validate its positive role in glucosinolate metabolism. Previously unidentified genes were assigned to this biosynthetic pathway and a regulatory network model was constructed [85].

Mao et al. [86] performed a gene co-expression network analysis of 1094 microarrays of Arabidopsis using a non-targeted approach. They identified 382 modules in this network. The top three modules with the most nodes were: photosynthesis, response to oxidative stress and protein synthesis. Many of the modules also involved responses to environmental stresses. They constructed a cold-induced gene network from a subset of microarrays. The response to auxin stimulus was the most over-represented of the 18 significant modules.

Carrera et al. [87] used the InferGene application to construct a regulatory model of the Arabidopsis genome. They used datasets from 1,486 microarray experiments. Ten genes were predicted to be the most central regulatory hubs influencing the largest number of genes. Included in this set were transcription factor genes involved in auxin (KAN3), gibberellin (MYB29), abscisic acid (MYB121), ethylene (ERF1), and stress responses (ANAC036). They computed the top 12 gene subnetworks; four of these were related to biotic and abiotic stresses. Eighty-five percent of the predicted interactions of the 25% most connected transcription factors were validated in AtRegNet, the Arabidopsis thaliana Regulator Network http://arabidopsis.med.ohio-state.edu/moreNetwork.html.

Lorenz et al [88] investigated the drought response of loblolly pine roots and identified a number of hubs in the transcriptional network. Highly ranked hubs included thioredoxin, an inositol transporter, cardiolipin synthase/phosphatidyl transferase, 9-cis-expoxycarotenoid dioxygenase, zeatin O-glucosyltransferase and a SnRK2 kinase. These genes are involved in phospholipid metabolism, ABA biosynthesis and signaling, and cytokinin metabolism; they appear to be important in stress mediation.

Weston et al [89] used weighted co-expression analysis to define six modules for Arabidopsis responses to abiotic stress. Two hubs in the common response module were an ankyrin-repeat protein and genes involved in Ca signaling. They created a compendium of genomic signatures and linked them to their co-expression analysis. Using the same approach, they extended their analyses to the responses of three different plant species to heat and light [90]. Species-specific responses were found involving heat tolerance, heat-shock proteins, ROS, oligosaccharide metabolism and photosynthesis.

## Time-series analyses reveal multiple phases in stress responses

Time-series analyses allow one to distinguish between primary and secondary responses to stress. In a comprehensive time-series transcriptomics analysis of 7 abiotic stresses on different Arabidopsis organs [28], a core set of genes (50% were transcription factors) of non-specific responses for all stresses were elucidated. Included in this set were the AZF2, ZAT10 and ZAT12 transcription factors. This initial response is thought to be involved in the readjustment of energy homeostasis in response to the stress. With time (after 1 h) more stress-specific profiles developed.

Sun et al [91] applied a complexity metric to a set of time series data of Arabidopsis with 9 different abiotic stresses. They found that genes with a higher complexity metric had longer 5' intergenic regions and a greater density of cis-regulatory motifs than the genes with a low complexity metric. Many of the cis-regulatory motifs identified were associated with previously characterized stress responses.

Vanderauwera et al. [92] investigated the effects of hydrogen peroxide (H_2_O_2_) signaling during high light stress using microarray analyses. They found that H_2_O_2 _was not only heavily involved in signaling in high light stress, but also salinity, water deficit, heat and cold stress. H_2_O_2 _was a key regulator of small and 70 kD heat shock proteins and many genes of the anthocyanin metabolic pathway. Anthocyanins appear to play an important role as antioxidants in plants. A specific UDP-glycosyltransferase (UGT74E2) was highly regulated by H_2_O_2_. In a subsequent study [93], UGT74E2 responded quickly to H_2_O_2 _and glycosylated indole-3-butyric acid (IBA) modifying auxin homeostasis, plant morphology and improving stress tolerance to salinity and water deficit. Furthermore, auxin was found to interact with ABA, increasing the ABA sensitivity of the plant. Silencing a poly(ADP-ribose) polymerase improved high light stress tolerance in Arabidopsis [94,95]. Part of the improved abiotic stress tolerance was ascribed to improved energy-use efficiency and reduced oxidative stress [94,95].

Kusano et al. [96] conducted a time-series experiment on the effects of UV-B light on Arabidopsis using both metabolomics and transcriptomics analyses. They found that plants responded in two phases with an upregulation of primary metabolites in the first phase and the induction of protective secondary metabolites, especially phenolics, in the second phase. The induction of phenolics corresponded to transcripts involved in the phenylpropanoid pathway, but the transcripts for primary metabolism were less consistent indicating that this pathway may be regulated by other mechanisms (e.g. kinases).

The transcriptomic response to drought can vary with the time of day [97]. These responses seem to interact with hormonal and other stress pathways that naturally vary during the course of the day. A smaller set of core genes were identified that responded at all times of the day. This set was compared to two previous studies and was whittled down to just 19 genes, including a NF-YB transcription factor, several PP2Cs, a CIPK7, and a sulfate transporter.

Drought stress studies and microarray analyses of three different genotypes of poplar clones grown in two different locations revealed epigenetic regulation to the environment [98]. The tree clones that had a longer history in the environment showed greater changes in DNA methylation, thereby influencing their response to drought.

Shoot tip growth of grapevines was found to be much more sensitive to osmotic stress than gene expression in a time-series experiment of the effects of gradual osmotic stress on grapevine [27]. Proteomics data indicated that changes in protein expression preceded and were not well correlated with gene expression (G.R. Cramer, unpublished results). The integration of transcriptomics data and metabolomics data indicated distinct differences of the responses of salinity and an isosmotic water deficit [27]. Drought-stressed plants induced greater responses in processes needed for osmotic adjustment and protection against ROS and photoinhibition. Salinity induced greater responses in processes involved in energy metabolism, ion transport, protein synthesis and protein fate. A comparison to similar short-term stresses [11] indicated that a gradual, chronic stress response was more complex than an acute stress response.

The effect of water-deficit on Cabernet Sauvignon berries (a red wine grape) in the field was studied using transcriptomics, proteomics and metabolomics [99-102]. Integrated analyses confirmed that the phenylpropanoid pathway (including anthocyanin and stilbene biosynthesis) was upregulated by water deficit in a tissue-specific manner in the skins of the berries. Other metabolic pathways in the berries were affected by water deficit including ABA, amino acid, carotenoid, lipid, sugar and acid metabolism. Most of these changes were associated with improved quality characteristics of the fruit.

Likewise, Zamboni et al. [103] investigated berry development and withering in grapevine at the transcriptomics, proteomics and metabolomics levels. A multistep hypothesis-free approach from four developmental stages and three withering intervals, with integration achieved using a hierarchical clustering strategy (multivariate O2PLS technique), identified stage-specific functional networks of linked transcripts, proteins and metabolites, providing important insights into the key molecular processes that determine wine quality. A hypothesis-driven approach identified transcript, protein and metabolite variables involved in the molecular events underpinning withering, which predominantly reflected a general stress response. Berry ripening and withering are characterized by the accumulation of secondary metabolites such as acylated anthocyanins, but withering also involves the activation of osmotic and oxidative stress response genes and the production of stilbenes and taxifolin.

Usadel et al. [104] investigated the effects of cold temperatures over time using transcriptomics, metabolomics and enzyme activities. They found some enzyme activities and metabolites changed rapidly, whereas others changed more slowly. The early changes (6 h) in enzyme activities were poorly correlated with transcript abundance, but after 78 h these correlations were greatly improved. Much of the long-term changes in metabolism could be ascribed to the CBF regulon.

Caldana et al. [105] conducted a complex time-series experiment (22 time points) with differing temperatures and light intensities using both metabolomics and transcriptomics analyses. This high-resolution time series experiment revealed that metabolic activities respond more quickly than transcriptional activities, indicating a disconnect between metabolism and transcription in the early phases of stress response and indicating that enzymatic activities may play a significant role. There were common metabolic responses to the changing environment within 1 h of the change including a decrease in energy metabolism and translation and an increase in the transcription of genes involved in signaling cascades. At later time points, condition-dependent metabolism was revealed. For example, protein degradation and energy metabolism derived from amino acids occurred in warm temperatures and darkness. Amino acid catabolism appears to fuel the TCA cycle in the absence of photosynthesis.

Yun et al. [106] characterized the response of rice to a mild chilling stress (10°C). They found that transcriptional regulation consisted of three dynamic and complex phases over 96 h. The early transcriptional phase appeared to be triggered by oxidative signals (H_2_O_2_) and lead to the subsequent induction of cellular defense and rescue mechanisms. Combining temporal co-expression data from microarrays with promoter motif enrichment analyses and oxidative responses, transcriptional regulatory network models for the different response phases were constructed. A bZIP-TGA transcription factor module (as1/ocs/TGA), one of seven transcription factor modules, was the most connected regulatory module in phase one. Each of the transcription factor modules consisted of clusters of transcription factors exhibiting combinatorial control of the chilling regulon. The speed of the response of this network was associated with chilling tolerance. Chilling-resistant genotypes had a much more rapid and pronounced response of this transcriptional regulatory network than chilling-sensitive genotypes. In addition, the transcription factors identified in this study were located within known growth and stress QTLs in the rice genome.

## Integration of omics analysis identifies molecular networks functioning in abiotic stress responses

Integrated omics analyses have markedly increased our understanding of plant responses to various stresses. These analyses are important for comprehensive analyses of abiotic stress responses, especially the final steps of stress signal transduction pathways.

Integrated analyses of the transcriptome and the metabolome successfully demonstrate connections between genes and metabolites, elucidating a wide range of signal output from ABA under dehydration [107] and the DREB1/CBF transcription factors in response to low temperature [108,109]. Metabolite profiling reveals that ABA accumulates during dehydration, regulating the accumulation of various amino acids and sugars such as glucose and fructose. In particular, the dehydration-inducible accumulation of BCAAs (branch-chain amino acids), saccharopine, proline, and agmatine are correlated with the dehydration-inducible expression of their key biosynthetic genes (*BCAT2*, *LKR/SDH*, *P5CS1*, and *ADC2*, respectively), which are regulated by endogenous ABA [107]. In addition, metabolome analysis of transgenic Arabidopsis overexpressing DREB1A/CBF3 reveals that there is a striking similarity between the low-temperature regulated metabolome (monosaccharides, disaccharides, oligosaccharides and sugar alcohols) and that regulated by the DREB1A/CBF3 transcription factor [108,109]. In particular, the low-temperature-inducible accumulation of galactinol and raffinose is correlated with the expression of the *Gols3 *gene, which is a direct target of DREB1A/CBF3 [108,109]. Maruyama et al. [109] also analyzed DREB2A overexpression, which did not increase the level of any low-temperature regulated metabolites in transgenic plants. Overexpression of DREB2A-CA in transgenic plants increased their tolerance to dehydration stress, but only slightly increased their tolerance to freezing stress [50]. These results indicate that the increased tolerance to freezing stress in transgenic plants overexpressing DREB1A may depend on the accumulation of low-temperature regulated metabolites, especially sucrose, raffinose, galactinol, and *myo*-inositol. Similarly, transcriptomics and metabolomics analyses of PSEUDO RESPONSE REGULATOR (PRR) arrhythmic triple mutant revealed that the DREB1A/CBF gene and raffinose amounts appear to be regulated by the circadian clock, varying between day and night as if in anticipation of the colder night temperatures [110].

Comparing metabolomics between dehydration, salinity, light, heat or low temperature stress have identified metabolites that are generally important in abiotic stress responses or are specific to each stress [27,95,105,111,112]. In a metabolite profiling study of Arabidopsis responses to combined dehydration and heat stresses [95], heat stress reduced the toxicity of proline, indicating that during the more severe combined stress treatment, sucrose replaces proline in plants as the major osmoprotectant. Comparative metabolite analysis between Arabidopsis responding to heat shock and cold shock revealed that the majority of metabolites in response to heat shock overlapped with those produced in response to cold shock [109,113]. These results indicate that a metabolic network of compatible solutes includes proline, monosaccharides (glucose and fructose), galactinol, and raffinose, which have an important role in tolerance to temperature stress. Wienkoop et al. [112] identified a RNA-binding protein (ATGRP7) that increased in response to low temperature stress and decreased in response to high temperature stress. Its abundance was significantly correlated with glutamine and proline concentrations. While raffinose and galactinol concentrations were significant markers for temperature responses, their response was independent of the responses of ATGRP7, proline and glutamine.

Transcriptomics, metabolomics and enzyme activities were integrated in a comprehensive study of K deficiency [114]. Carbon and nitrogen metabolism were significantly affected by K deficiency. This integrated approach pinpointed that pyruvate kinase activity (not transcription) was inhibited directly by K deficiency and was primarily responsible for the metabolic disorders observed.

## Systematic application of omics technologies has contributed to the development of stress-tolerant crops in the field

Many genes affect stress tolerance, but few of the identified genes have proven useful in the field. Due to the complexity of stress interactions and stress responses, relevant phenotyping needs to be performed (including field experiments) in abiotic stress studies if we are to make significant progress [113]. The following studies are discussed to highlight good examples of systems biology and omics approaches that have been used to identify key genes regulating stress tolerance and then followed with validation of those responses and phenotypes in multiple experiments including field conditions.

A SNAC1 gene was identified from microarray experiments of stress treatments on rice [115]. SNAC1 is a NAC transcription factor that induces the expression of a number of stress-tolerance genes and improves the drought and salt tolerance of rice in the field. The transgenic plants exhibited increased sensitivity to ABA and reduced water loss. In another drought stress study, a LEA (late embryogenesis abundant) gene was identified from microarray experiments of rice and was transformed and tested in the field under drought conditions through the T3 generation [116]. Spikelet fertility appears to be the main factor contributing to improved yields under drought conditions.

An exhaustive screen of greater than 1500 transcription factors in Arabidopsis identified approximately 40 transcription factors that when overexpressed, improved stress tolerance [117]. One of these transcription factors NF-YB1 was further characterized and shown to display significant drought tolerance in Arabidopsis. Microarray data of this overexpressing line showed few differences in gene expression and the genes identified were not known previously to be involved in drought tolerance. This functional genomics approach provided a new strategy for improving drought tolerance in plants. A homolog of NF-YB1 was cloned in maize (ZmNF-YB2), overexpressed and tested for drought tolerance in the greenhouse and field plots. The transgenic maize lines were more drought tolerant having increased chlorophyll content, photosynthesis, stomatal conductance and grain yields. One line consistently had more than 50% yield improvement in drought conditions over two different years.

Oh et al. [118] used microarrays to identify 42 AP2 transcription factors whose expressions were affected by stress. Two of these transcription factors, AP37 and AP59 were functionally characterized. The two transcription factors are closely related but have distinct differences in affecting rice phenotype. AP37 responded to drought, salinity, cold and ABA; over-expression improved stress tolerance to all three environmental conditions. AP59 responded to drought and salinity, but not cold or ABA, and improved stress tolerance to drought and salinity only. Both overexpressing lines showed improved photosynthetic efficiency under stress conditions. Overexpression of the transcription factors induced common and distinct sets of genes. T5 homozygous overexpressing lines of AP59, but not AP37, had yield penalties under normal paddy conditions in the field, whereas AP37 overexpressing lines, but not AP59, had enhanced yields under drought conditions in the field. The reduced yields of the overexpressing lines of AP59 were attributed to effects on spikelet development. This study emphasizes the point that it is important to characterize gene effects on yield under field conditions.

## Mapping stress responses has provided new insights and identified gaps in our knowledge of abiotic stress responses

From a meta-analysis of drought-stress related papers from the last 15 years, a complex model for plant responses to drought stress was produced [12]. This model details the interactions of sugars, ROS/RNS, hormones (ABA, ethylene, auxins, cytokinins, salicylic acid, gibberellin and brassinosteroids) and nitrogen metabolism. It highlights the highly complex nature of stress responses.

From this review, we have constructed a simplified working model summarizing some of the known plant signaling responses to abiotic stress (Figure 2). Much of the signaling involves phosphorylation cascades that react quickly in the plant cell, emphasizing the need for proteomics data as well as transcriptomics data in future models. The PYR/PYL/RCAR-PP2C-SnRK2 pathway illustrates that protein phosphorylation and dephosphorylation are the most important factors in ABA signaling. Similar phosphorylation and dephosphorylation processes are involved in ethylene and other abiotic stress signaling pathways (Figure 2). Not all connections could be drawn in this two-dimensional figure without obscuring many other connections. For example, the interactions of ROS with abiotic stresses and hormones [32] are too complex to display here. In addition, the actual signaling response will be dependent upon the signaling pathway present in that organ, tissue or cell at the time of the response. One needs to use more sophisticated bioinformatics programs like Cytoscape [119] and its plug-ins to visualize the interactions comprehensively in two dimensional or three-dimensional space [120] or with time series views [121], which would allow these data to be viewed in four dimensions.

Although there are still some technological issues that must be solved to produce a complete picture of protein phosphorylation, several technologies have been developed for the large-scale analysis of phosphoproteins, known as 'phosphoproteomics' [122]. Mass spectrometry analyses have identified thousands of phosphoproteins in Arabidopsis, rice, and *Medicago truncatula *[123-125]. In addition, two studies have reported ABA-responsive changes in the phosphoproteome [126,127]. Phosphoproteomics analyses of mutants for abiotic stress signaling (e.g. PP2C or SnRK) will identify the relevant network of protein phosphorylation events in abiotic stress signaling.

Transcriptome analysis technologies have advanced to the point where high-through-put DNA sequencers and high-density microarrays such as tiling arrays are readily available. These technologies provide new opportunities to analyze noncoding RNAs and can clarify aspects of epigenetic regulation of gene expression [128,129]. Similar approaches [130,131] have revealed the global transcriptomes of plants exposed to abiotic stresses such as dehydration, cold, heat, high-salinity, osmotic stress, and ABA. These analyses indicate that these stresses increase or decrease transcript abundance from not only previously identified stress-responsive genes, but also from thousands of unannotated non-protein-coding regions. Matsui et al. [130] estimated that approximately 80% of previously unannotated upregulated transcripts arise from antisense strands of sense transcripts. There was a significant linear correlation between the expression ratios (stress-treated/untreated) of the sense transcripts and the ratios of the antisense transcripts. Interestingly, the data suggested that such stress-responsive antisense transcripts are derived from antisense strands of the stress-responsive genes, *RD29A *and *CYP707A1*. Clearly, transcriptional regulation is far more complicated than we previously imagined. Whether or not such antisense transcripts have biological functions is an important issue that remains to be resolved.

Much more research is required in order to fully map plant responses to abiotic stress. The nature of the pathway responses will vary and is highly dependent on the species, organ, tissue, cell type, developmental stage of the plant, the stress or stresses affecting the plant, the level and duration of the stress. Despite the vast amount of research collected on abiotic stress in the last decade, there are still significant gaps in our knowledge. We still do not understand completely how plants perceive stress. We don't know all of the receptors and their sites of action (organs, tissues and cellular components). While we know a lot about downstream signaling (i.e. transcriptional pathways), we know very little about the primary signaling (i.e. proteomics). Most of the literature on abiotic stress responses in plants is based upon transcriptomics data rather than proteomics data. This is not surprising as transcriptomics technology is more advanced, easier to perform and less expensive. However, transcriptomics analyses are insufficient as there is an overall poor correlation of transcriptomics profiles with proteomics profiles [101,132,133] or enzyme activities [104,114]. There are only a few studies describing phosphorylation cascades and other post-translation modification activities in response to stress [134]. Recent efforts to map the hormone [126,127] and light-regulated [135] phosphorylomes are good first steps. Finally, we need better tools to facilitate systems biology analyses especially in the area of bioinformatics. Transcriptomics data can be collected in a matter of days or weeks, but the data analyses often take more than a year.

## Conclusions

We have made great progress in understanding the responses of plants to abiotic stress. There are inherent physical, morphological and molecular limitations to the plant's ability to respond to stress. Systems biology approaches have given us a more holistic view of the molecular responses. Transcriptomics studies are well advanced, but proteomics analyses are lagging behind, especially the study of post-translational modifications. Plant responses to abiotic stress are dynamic and complex. The integration of multiple omics studies has revealed new areas of interactions and regulation. Time series experiments have revealed the kinetics of stress responses, identifying multiple response phases involving core sets of genes and condition-dependent changes. One consistent trend in response to abiotic stress is the early down regulation of energy metabolism and protein synthesis. This may indicate a conservation of energy by the plant and may reflect a shift from plant growth to protective mechanisms. In many examples presented in this review, ABA signaling mediates the plant responses to abiotic stress. Co-expression analyses are useful in that they have revealed key regulatory hubs that can be manipulated to produce different phenotypes. To get a comprehensive understanding of plant responses to abiotic stress, more extensive mapping of these responses at the organ, tissue and cellular level are needed. Such network analyses need to be extended to the proteomics and enzyme activities levels. Models need to be constructed and linked to phenotypic traits. The linkage of key regulatory hubs to phenotypic traits will allow for more rapid progress in the genetic manipulation and production of crop plants. Current progress is exemplified by the identification and validation of several key genes that improved stress tolerance of crops in the field. It is expected that progress in the plant sciences and systems biology will continue to accelerate in the near future.

## Authors' contributions

GRC contributed to all aspects of this manuscript. KU and KS contributed to the metabolomics and ABA signaling sections and Figure 2. SD contributed to the introduction, ABA and sugar signaling sections. MP contributed to the time-series analyses section. All authors read and approved the final version of this manuscript.
